# Endo-periodontal lesion – endodontic approach

**Published:** 2014

**Authors:** R Jivoinovici, I Suciu, B Dimitriu, P Perlea, R Bartok, M Malita, C Ionescu

**Affiliations:** * Department of Endodontics, Faculty of Dental Medicine, “Carol Davila” University of Medicine and Pharmacy, Bucharest; ** Department of Conservative Dentistry, Faculty of Dental Medicine, “Carol Davila” University of Medicine and Pharmacy, Bucharest; *** Faculty of Midwives and Medical Assistants, “Carol Davila” University of Medicine and Pharmacy, Bucharest; **** Faculty of Dental Medicine, “Carol Davila” University and Medicine and Pharmacy, Bucharest

**Keywords:** Endo-periodontal lesion, endodontic, sulcus, canal

## Abstract

Endo-perio lesions might be interdependent because of the vascular and anatomic connections between the pulp and the periodontium.

The aim of this study is to emphasise that primary endodontic lesion heals after a proper instrumentation, disinfection and sealing of the endodontic space.

The primary endodontic lesion with a secondary periodontal involvement first requires an endodontic therapy and, in the second stage, a periodontal therapy. The prognosis is good, with an adequate root canal treatment; it depends on the severity of the periodontal disease, appropriate healing time and the response to the treatment.

A correct diagnosis is sometimes difficult; an accurate identification of the etiologic factors is important for an adequate treatment.

Primary perio-endo lesion may heal after a proper disinfection and sealing of the endodontic system, the one-year follow-up radiograph showing bonny repair. Invasive periodontal procedures should be avoided at that moment.

The microorganisms and by-products from the infected root canal may cross accessory and furcal canals and determine sinus tract and loss of attachment. In both clinical cases presented in this article, successful healing was obtained after a proper disinfection and sealing of the endodontic system.

## Introduction

A retrospective study of Blomlof [**4**] concluded that the endodontic infection promotes periodontal pocket formation and is a risk factor in the progression of periodontitis, so, a primary endodontic lesion draining through the attachment apparatus should be initially treated by an endodontic treatment [**6**], since an aggressive removal of the periodontal ligament and cementum during the endodontic therapy, adversely affects periodontal healing [**3**,**4**].

**Case report 1**

**Fig. 1:**
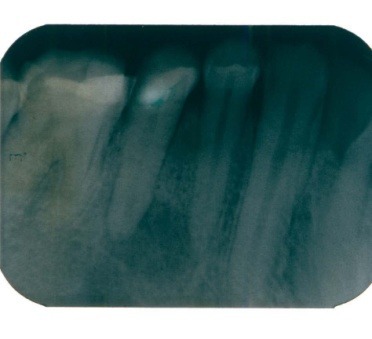
Tooth 45 with enlargement of the periodontal space, apical and lateral radiolucency

A 35-year-old patient presented with tooth 45 presenting symptomatic swellings of the soft tissue, probe depths were in normal limit, except for one area on the distal aspect (8 mm) and pus drained through the sulcus. A periradicular severe bone loss and a lateral mesial were observed at the radiographic examination (**Fig. 1**).

**Fig. 2:**
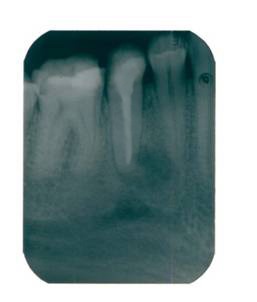
Radiograph after conventional endodontic treatment

**Fig. 3:**
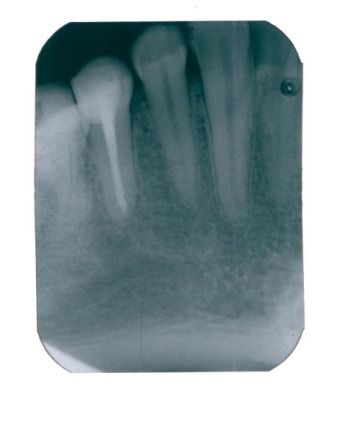
Follow-up radiograph, one year after root canal therapy, the resolution of the periradicular bone lesions is evident

After the instrumentation, an interappointment dressing with calcium hydroxide was used. After four weeks, the tooth was asymptomatic (**Fig. 2**), no pus was draining in the sulcus, the canal was filled with lateral condensation, gutta-percha and sealer and coronal restoration. After 2 years (**Fig. 3**) the complete resolution of the radiolucency was observed and probing depths were of 3 mm around the tooth.

The radiographic appearance of tooth 45 (**Fig. 1**) was performed on the initial presentation with one month after the conventional endodontic treatment (**Fig. 2**).

After the appropriate endodontic approach, the periodontal treatment was performed (upper and lower gingival scaling, root planning and dental deep cleaning).

**Case report 2**

A 53-year-old patient presented with a swelling of the buccal aspect of the mucosa of teeth 36 and 37, symptomatic with probing depths of 16 mm of the buccal and mesial surface of the teeth.

Clinically, a deep disto-buccal periodontal defect could be probed.

**Fig. 4:**
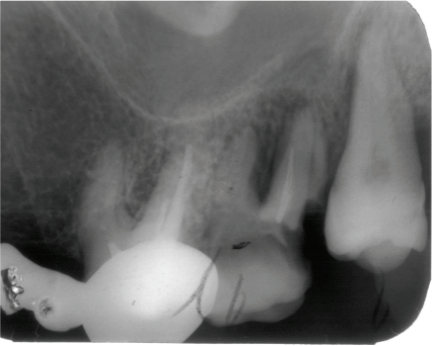
Preoperative radiograph indicates bone loss around the apices of 27 and furcal radiotransparency 26 and 27

**Fig. 5:**
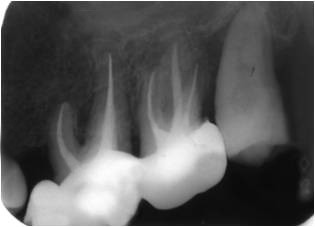
One year follow up radiograph showing resolution of most of the periradicular lesions

Clinically, the buccal defect healed after one year and the pocket probing depth was normal, except for the mesial aspect of 26 and distal aspect of 27.

**Fig. 6:**
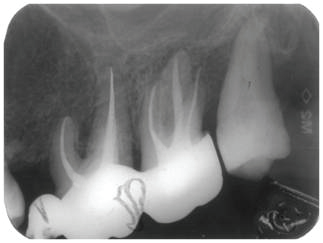
However, a bonny defect at the furcal area was still present and also a solitary pocket extended towards the apex was found regarding the mesial aspect of 26 and distal aspect of 27

The root canal was instrumented by using nickel titanium rotary instrumentation and calcium hydroxide was placed as an interappointment dressing. After three weeks, the endodontic system was filled with a lateral condensation of gutta percha. At the follow-up examination, one year later, the probe depths were 3 mm all around the tooth, except for the mesial aspect (14mm). The follow-up radiographic examination after two years showed a regeneration of the periradicular tissue with persistence in teeth 27, 26 of deep furcal defect extended to the apex of the mesial root.

After the root canal treatment was completed, periodontal therapy was initiated.

The endodontic treatment alone did not influence the complete healing of the defect, a periodontal treatment was necessary for the further healing of the furcal area and inflamed gingival tissue (upper and lower gingival scaling, root planning and open field periodontal curettage).

## Discussions

Primary endodontic lesions with secondary periodontal involvement, primary periodontal lesions with secondary endodontic involvement and combined lesions are difficult to differentiate clinically and radiographically; where doubt exists, it should be considered as an endodontic lesion [**1**,**2**].

The primary endodontic disease with secondary periodontal involvement should first be treated with an endodontic therapy. The treatment results should be evaluated after two or three months and only then, should the periodontal treatment be considered. Prognosis depends on the severity of the periodontal involvement, periodontal treatment and patient response [**5**].

The size of the periapical lesion in an isolated endodontic disease does not affect the clinical outcome [**10**].

The differential diagnosis is difficult when a sinus tract, originating from the endodontic lesion may drain along the periodontal ligament, giving the appearance of periodontal disease [**7**,**9**]. Therefore, a primary endodontic lesion draining from the attachment apparatus should be initially treated by an endodontic therapy [**10**]. Periradicular lesions may initially expand horizontally through cancellous bone and then proceed vertically [**8**].

## Conclusion

1. Because the endodontic pathogens and their byproducts may affect the integrity of the periodontium, they have to be eliminated during root canal treatment.

2. The placement of calcium hydroxide paste is indicated, because of its antibacterial, proteolytic and anti-inflammatory proprieties, it might inhibit resorption and periodontal contamination, before the periodontal treatment is initiated.

3. If proper endodontic treatment is performed, sinus tract extending to the gingival sulcus or furcation area can heal.

4. Usually, the endodontic treatment alone is not sufficient for healing the combined lesion and a proper periodontal treatment is needed to influence the secondary periodontal disease.
